# Think Smooth and Pink: The Role of Skin Color and Texture in Caucasian Female Genital aesthetics

**DOI:** 10.1093/asj/sjaf230

**Published:** 2025-11-07

**Authors:** Marek Jankowski, Michał Buszka, Oliwia Bąkowska, Aleksandra Tołopiło

## Abstract

**Background:**

Female genital cosmetic surgery (FGCS) is increasingly popular; yet, research has largely focused on labia size while neglecting the estrogen-related color and quality of genital skin. Evidence from facial attractiveness studies and comparative primatology suggests that genital skin perfusion and smoothness may contribute to perceived attractiveness.

**Objectives:**

We investigated whether vulvar skin perfusion and smoothness predict perceived genital attractiveness and compared its impact with that of labia size and volume.

**Methods:**

A standardized image database of 1080 vulvar images from healthy women was analyzed for morphometric and colorimetric features. Representative images (n = 360) were digitally manipulated to vary skin perfusion (measured as a* value). Attractiveness judgments were obtained from 159 Polish adults (45 men, 114 women; mean age = 26.7) in 40,991 forced-choice paired comparisons. Bradley–Terry models estimated latent attractiveness scores. Analyses of covariance tested chromatic manipulations, and hierarchical regression identified predictors across colorimetric, morphological, and demographic factors.

**Results:**

Enhanced vulvar skin perfusion significantly increased attractiveness relative to natural and reduced skin perfusion (*P* < .001, η*_p_*² = .17). The regression models explained 59% of the variance. Positive predictors included skin redness, greater apparent labia majora volume, and smooth skin texture. The negative predictors were increased labia minora visibility, skin wrinkling/atrophy, presence of a supralabial crease, lower skin lightness, greater skin yellowness, and model age.

**Conclusions:**

Perceived genital attractiveness is shaped by both chromatic and morphological features, with vulvar skin perfusion and texture emerging as important determinants. These findings suggest that FGCS planning should look beyond labiaplasty alone, incorporating interventions targeting skin color and quality.

**Level of Evidence: 5 (Therapeutic):**

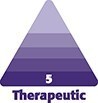

The interest in and demand for female genital cosmetic surgery (FGCS) are steadily increasing.^1^ The term encompasses various techniques aiming to ameliorate the female vulvar area functionally and to alter it aesthetically. These various interventions for the female vulva can include labia majora augmentation by fillers or autologous fat transplantation, labial depigmentation by chemical peels or by laser, mons pubis reduction by liposuction, and reduction of the labia minora size, known as labiaplasty. The latter is currently the most popular FGCS procedure, and the concept that the labia minora should be covered by the labia majora, which must converge and partially cover the clitoris, has been internalized by surgeons^2^ and considered the societal ideal.^3^ The anatomical criteria behind these interventions are debatable, as the female vulva shows significant diversity. Morphometric analysis revealed significant variation and a normal distribution of labia minora length and width.^4-8^ Age, race, BMI, height, and parity did not influence labia size, although one study reported wider labia minoras in premenopausal women than in postmenopausal women.^8,9^ In contrast to the aforementioned FGCS standard, protruding and easily visible labia minora are more common than not,^5-7^ with asymmetry reported in up to 42.5% of women not seeking FGCS.^10^ Among women with protruding labia minora, only 7.3% considered their labia to be abnormal.^6^ Moreover, among labiaplasty patients, the degree of protrusion of the labia minora does not correlate with the degree of complaints^5,11^ or sexual function.^12^ In fact, the component of genital self-image derived from labia minora proportions is culturally influenced. Labia minora elongation treatments are popular across some ethnic backgrounds,^13,14^ and exposure to images of surgically modified vulvas was shown to affect women's perceptions of desirable labia proportions.^15^

Although the influence of the geometric properties of the vulva on attractiveness has been thoroughly studied, little attention has been given to the chromatic properties of vulvar skin. Nonetheless, treatments affecting genital skin color have become increasingly popular and are associated with high patient satisfaction.^16-19^ Among females considering FGCS, 24% are in fact dissatisfied with their vulvar color.^20^ Comparative primatology provides a rationale for greater interest in the chromatic properties of vulvar skin. In at least nine genera of catarrhine primates, including the closest human relative, chimpanzee, the coloration of the female genital area is involved in sociosexual signaling.^21,22^ Depending on the species, some extent of the perigenital area, referred to as “sexual skin,”^23^ has a more reddish or pinkish hue, which results from its vascular specialization and its control by hormonal factors.^24^ In rhesus monkeys, reddening of facial, nipple, and genital skin constitutes a major signal of attractiveness during the mating period,^25^ whereas iatrogenic menopause leads to fading of the red coloration of genitalia, which is restored by exogenous estrogens.^26^ In humans, the expression of estrogen receptors has been demonstrated in keratinocytes and fibroblasts from vulvar and perineal skin but not in those from extragenital sites.^27^ By analogy to other primate species, we hypothesize that chromatic variation in female genital skin is a valid cue of perceived aesthetic attractiveness. Specifically, we predict that the perception of attractiveness is positively correlated with the estrogen-dependent concentration of oxyhemoglobin in the skin (skin perfusion), which is reflected by higher a* values in the CIELAB color space. The general aim of this study was to explore the relationship between the skin tone of the vulva and perceived genital attractiveness and to examine how much vulva skin color affects perceived attractiveness compared with vulva morphological features. A better understanding of the impact of chromatic properties of genital skin on the perception of attractiveness can be utilized to optimize decision making and planning of (or discouraging) any genital rejuvenation and beautification surgery.

## METHODS

### Image Database

An image database was sourced from a leading adult media company. Adult media databases were previously shown to be valid sources of anthropometric data.^28^ The database consists of casting photos of 2073 adult individuals aged 18-68 years taken with the same lighting setup. A total of 1383 entries included high-resolution images of the models' vulvas. Images with conspicuous pubic hair were removed, leaving 1080 images for anthropometric analysis. Geometric measures such as the area of the vulva and the visible area of the labia minora were measured with ImageJ (National Institutes of Health, Bethesda, Maryland, USA). As absolute labia minora length is not an easily available cue in visual inspection, we used the visible labia minora area as a proxy for size. To exclude possible bias resulting from differences in camera zooming, only the relative values of the visible area of the labia minora to the area of the vulva were further analyzed. Vulva skin texture was classified on a 4-point scale: 0—smooth skin, no rugosity perceptible, 1—perceptible rugosity, scrotal appearance but not affecting the contour, 2—visible deformation of the labia contour, slight atrophy, 3—conspicuous deformation of the labia contour, and advanced skin atrophy. The apparent labia majora volume was classified on a 4-point scale: 0—labia majora does not form a visible skin fold, following the curvature of the adjacent skin; 1—labia majora form a visible skin fold with concave curvature; 2—labia majora form a visible skin fold with convex curvature; and 3—labia majora form a thick skin fold with convex curvature. The width of the labia is at least one third of their length. Apparent volume classification was performed in the context of images presenting models with legs spread 90° apart. The “supralabial fold” feature was considered positive if there was a clearly visible skin crease between the labia and the mons pubis.

Images were converted to the CIELAB color scale in Adobe Photoshop (Adobe, San Jose, California, USA); subsequently, color values were measured with a 101 × 101-pixel sampling brush from the following genital skin: anterior comisura labiorum majorum, labia majora and labia minora mid-length, and from the groin as a reference for nongenital skin. Color contrasts from standardized locations^29^ were measured as previously described.^30^ Positive L* values indicate that the feature skin is lighter than the surrounding skin, whereas positive a* values indicate that the surrounding skin is redder than the feature.

### Image Manipulation

Representative vulvar images (n = 360) that were homogeneously distributed according to the chromatic properties and anatomical proportions were sourced from the aforementioned database. Multiple natural photographs were used instead of morphed images to retain the ecological validity of the study. We used Photoshop to produce a mask on the vulva with Gaussian blur at the edges to conceal manipulation. We manipulated the vulva color on the CIELAB a* (redness) color axis by +10 or −10 units, creating sets of 3 images with equal morphology but differing in redness, which gave us a set of 1020 stimulus images. Although the precise threshold is context dependent, data from facial stimuli imply that this is a suprathreshold difference.^31^ The lightness and yellowness of the skin, as well as the rest of the images, remained constant.

### Attractiveness Survey

A set of images was presented to the respondents in a forced choice scenario with 2 randomly selected images presented together. The respondents were asked to choose the most attractive image with no time limit. The preferred images were randomly paired again until one, and the most preferred image remained. We chose image comparisons instead of ratings to minimize the bias effect of different mental scales of participants. Different chromatic versions of this same image were never shown together to conceal the purpose of the study. Image presentation was balanced between the left and right visual fields. The survey was conducted between November 2024 and March 2025.

## RESULTS

### Image Ranking

The study involved 159 Polish, Caucasian adults (45 men, 114 women) aged 18-70 years (*M* = 26.7, *SD* = 7.2), who provided a total of 40,991 pairwise image comparisons. Given that participants' paired comparisons select the more attractive image, a Bradley–Terry model^32^ was used to estimate latent attractiveness for each image. This probabilistic model effectively addresses paired comparison data by evaluating relative preferences and generating continuous attractiveness estimates. On the basis of these scores, the images were ranked from most to least attractive.

### Effect of Increased Vulvar Skin Perfusion (Redness) on Perceived Attractiveness

**Figure 1.**
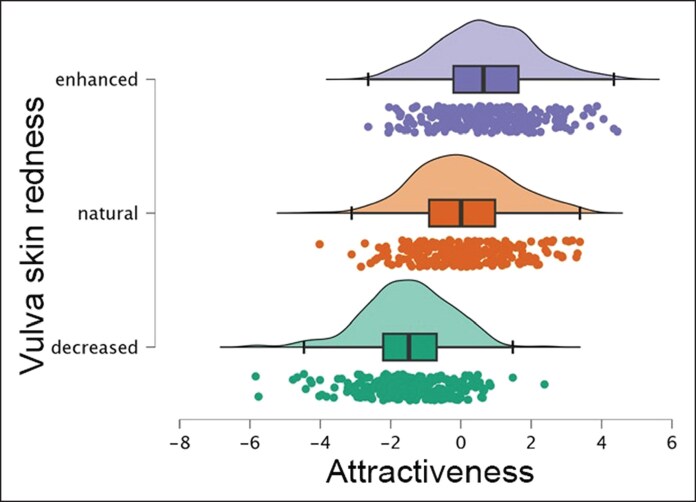
Raincloud plot showing the distribution of attractiveness ratings across 3 experimental conditions of vulva skin redness.

To examine differences in attractiveness ratings across the 3 experimental conditions (decreased vs natural vs enhanced genital skin perfusion), we conducted one-way analyses of covariance (ANCOVAs) while statistically controlling for the model's age and *value of the vulva. Owing to the large number of variables, all colorimetric and contrast-based indicators were computed as the mean values for the whole vulva area derived from both the labia majora and labia minora measurements. There was a significant main effect of condition on attractiveness ratings, *F*(2, 764) = 80.61, *P* < .001, η*_p_*² = .17, as well as a significant covariate effect of age, *F*(1, 764) = 46.82, *P* < .001, η*_p_*² = .06, and a significant covariate effect of *a coordinate, *F*(1, 764) = 75.18, *P* < .001, η*_p_*² = .09. Post hoc comparisons with Holm correction revealed significant differences across all conditions (*Ps* < .05; see Table 1 for detailed statistics). Marginal means showed that vulva images with decreased skin perfusion (*M* = –1.16, *SE* = 0.09) were rated the least attractive, followed by those with natural color (*M* = 0.10, *SE* = 0.08) and enhanced skin perfusion (*M* = 0.38, *SE* = 0.09), which were perceived as the most attractive (Figure 1). Thus, the manipulation of *a coordinate of vulvar skin (which is naturally dependent on estrogen-controlled skin vascularization) had a significant effect on attractiveness ratings.

**Table 1.** Pairwise Post Hoc Comparisons for Vulva Skin Redness Preferences

		*t*	*df*	*pHolm*	*Cohen's d*
Natural	Enhanced	2.46	764	.038	0.23
	Decreased	10.97	764	<.001	1.04
Enhanced	Decreased	11.59	764	<.001	1.28

### Identifying Key Predictors of Vulvar Attractiveness: A Stepwise Regression Analysis

#### Preliminary Correlation Analysis

Before conducting the regression analysis, Pearson's correlations were computed to examine relationships among predictors of attractiveness and to assess multicollinearity. Several moderate to strong correlations were observed, such as between CIELAB *a and *b vulva color coordinates (*r* = .46, *P* < .001) and a strong negative correlation between absolute color values and Michelson color contrast (*r* = −.81, *P* < .001), raising concerns about potential collinearity. On the basis of these results, all Michelson contrasts were excluded from subsequent regression models to mitigate multicollinearity. All remaining predictors were retained for hierarchical analysis, with variance inflation factors (VIFs) monitored throughout model building. In contrast, age was weakly or nonsignificantly correlated with other variables, suggesting that it may account for unique variance.

### Model-Building Strategy

To identify the key predictors of attractiveness ratings, we employed hierarchical stepwise linear regression analysis incorporating colorimetric, contrast-based, morphological, and skin texture characteristics of the vulva. Four nested models were constructed sequentially.

Model 0 served as the baseline with no predictors. Model 1 included the vulva skin color (CIELAB *L, *a, *b coordinates) and color contrast (Δ*E*) between the vulva and surrounding nongenital skin. Model 2 included additionally the following morphological features of the vulva: visible area of the labia minora, apparent labia majora volume, labia majora skin smoothness, and the presence of a skin crease between the mons pubis and the labia majora. Finally, Model 3 added age as a covariate to control for its potential confounding influence.

#### Model Fit and Variance Explained

Each successive model demonstrated a statistically significant improvement in model fit, as shown by increases in *R*² and decreases in the RMSE, AIC, and BIC. Model 1 accounted for 36.3% of the variance in attractiveness ratings (*R*² = .36, RMSE = 1.32), whereas Model 2 improved this value to 56.3% (*R*² = .56, RMSE = 1.10). The final model (Model 3) explained 58.5% of the variance (*R*² = .59, RMSE = 1.07). ANOVA comparisons confirmed that each model significantly improved the fit compared with the previous model (*P* < .001).

#### Significant Predictors in the Final Model

In the final model, several variables emerged as statistically significant predictors of attractiveness ratings. Among the color-related features, the *a (skin redness, perfusion) coordinate of the vulva was the strongest positive predictor (*b* = 0.22, *SE* = 0.01, *t*[768] = 25.33, *P* < .001), indicating a preference for redder labial pigmentation. The *L (lightness) and *b (yellow–blue) coordinates were both significant negative predictors (*b* = −0.02, *SE* = 0.01, *t*[768] = 3.84 and *b* = −0.05, *SE* = 0.01, *t*[768] = 3.88, respectively; *Ps* < .001), suggesting a preference for lighter and less yellow labial hues. However, in the fully adjusted model, the global color contrast between the vulva and nongenital skin (Δ*E*) was not a significant predictor (*Ps* > .05), suggesting that once pigmentation, morphology, and age were accounted for, this contrast measure did not substantially contribute to attractiveness evaluations.

Among the morphological features, the visibility of the labia minora remained a significant negative predictor (*b* = −1.54, *SE* = 0.43, *t*[768] = 3.56, *P* < .001), confirming that less visible labia minora were rated as more attractive. Higher grades of apparent labia majora volume were associated with significantly higher attractiveness ratings relative to the least voluminous labia, with the strongest effect for the most voluminous category (*b* = 0.93, *SE* = 0.16, *t*[768] = 5.89, *P* < .001). In contrast, labia skin texture emerged as a strong negative predictor. Compared with the reference smooth skin, more wrinkled skin textures were associated with significantly lower ratings, with the largest negative effect observed for labia with apparent atrophy (*b* = −2.19, *SE* = 0.22, *t*[768] = 10.19, *P* < .001). Moreover, the presence of a skin crease between the labia majora and the mons pubis was associated with lower attractiveness (*b* = −1.03, *SE* = 0.14, *t*[768] = 7.20, *P* < .001).

The model's age was a significant negative predictor (*b* = −0.03, *SE* = 0.01, *t*[768] = 6.94, *P* < .001), reflecting a robust preference for younger models.

### Effects of Categorical Predictors of Labia Attractiveness: Analyses of Covariance

To examine differences in attractiveness ratings across morphological categories, we conducted 3 separate 1-way ANCOVAs. Each analysis assessed the effect of a specific categorical factor—labia skin texture, labia apparent volume, and presence of the supralabial skin crease—while statistically controlling for the model's age. All 3 factors significantly influenced perceived attractiveness, although the size of the effects varied. Labia skin texture accounted for the largest portion of explained 12% of the variance (η*_p_*² = .12), followed by apparent labia majora volume, which explained 6% of the variance (η*_p_*² = .06), and the presence of the supralabial fold, which explained 3% (η*_p_*² = .03). Age was a significant covariate in all the models, explaining between 4% and 7% of the variance.

#### Labia Majora Skin Texture

**Figure 2.**
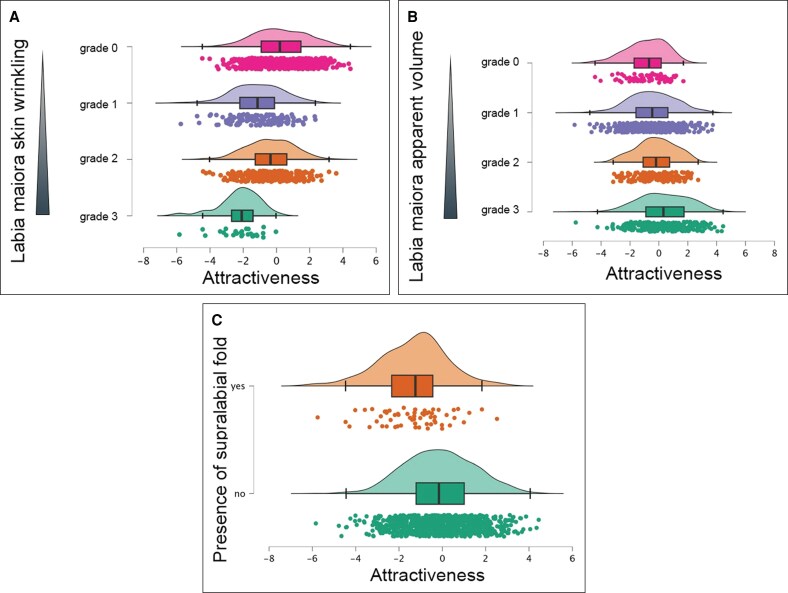
(A) Raincloud plot showing the distribution of attractiveness ratings across labia maiora skin texture categories. (B) Raincloud plot showing the distribution of attractiveness ratings across apparent labia maiora volume categories. (C) Raincloud plot showing attractiveness ratings based on the presence or absence of a supralabial fold.

There was a significant main effect of labia skin texture on attractiveness ratings, *F*(3, 896) = 40.32, *P* < .001, η*_p_*² = .12, as well as a significant covariate effect of age, *F*(1, 896) = 38.32, *P* < .001, η*_p_*² = .04. Post hoc comparisons with Holm correction revealed significant differences between all categories (*P* < .01; see Supplemental Table 1, available online at https://doi.org/10.1093/asj/sjaf230 for detailed statistics). Marginal means showed that Grade 3 (atrophic skin) (*M* = −1.96, *SE* = 0.29) was the least attractive, followed by Grade 2 (*M* = −1.03, *SE* = 0.13), Grade 1 (scrotal appearance) (*M* = −0.39, *SE* = 0.09), and Grade 0 (smooth skin) (*M* = 0.23, *SE* = 0.07), which was perceived as the most attractive (see Figure 2A).

#### Labia Majora Apparent Volume

A significant main effect of labia bulkiness on attractiveness was observed, *F*(3, 895) = 20.07, *P* < .001, η*_p_*² = .06, as was a significant covariate effect of age, *F*(1, 895) = 69.32, *P* < .001, η*_p_*² = .07. Post hoc comparisons with Holm correction revealed significant differences between most bulkiness categories, except concave vs convex categories, which did not differ significantly (*P* > .05; see Supplemental Table 2, available online at https://doi.org/10.1093/asj/sjaf230 for detailed statistics). The most voluminous labia category (Grade 3) received the highest attractiveness ratings (*M* = 0.37, *SE* = 0.10), followed by Grade 2 (*M* = −0.31, *SE* = 0.12), Grade 1 (*M* = −0.40, *SE* = 0.08), and the least voluminous Grade 0 (*M* = −1.01, *SE* = 0.20), which was rated the least attractive (see Figure 2B).

#### Supralabial Skin Crease

There was a significant main effect of supralabial skin crease on attractiveness, *F*(1, 898) = 30.19, *P* < .001, η*_p_*² = .03, as well as a significant covariate effect of age, *F*(1, 898) = 60.24, *P* < .001, η*_p_*² = .06. Post hoc comparisons with Holm correction indicated that the absence of supralabial crease was rated significantly more attractive (*M* = −0.13, *SE* = 0.05) than its presence (*M* = −1.26, *SE* = 0.20), *t*[898] = 5.50, *P* < .001, Cohen's *d* = 0.72 (see Figure 2C).

## DISCUSSION

This study enhances knowledge base by providing first objective data in the underexplored area of genital skin quality. The results of this study clearly demonstrated a significant positive correlation between genital attractiveness and skin perfusion, which has previously been found to be positively correlated with female facial attractiveness and healthiness.^33^ Facial skin perfusion, which is dependent on oxyhemoglobin concentration, is known to correlate with aerobic fitness, thus indicating generally better fitness.^34^ However, it is unclear whether general fitness is relevant in the context of genital skin coloration. Since estrogen receptors are present in human vulvar skin^27^ and estrogen-dependent genital skin reddens signals potential fertility in nonhuman primates, our alternative hypothesis is that a more reddish hue is a cue to age and fertility status in humans as well and, because of that, is perceived as attractive.

Our findings contradict the results of Johns et al,^35^ who argue that heterosexual men do not prefer redder female genitalia. The subset of images in our dataset was rated lower after increasing vulva perfusion than the original images were rated, suggesting that a cap effect perfusion has on attractiveness. The images used in their study came from young women, and they had high a* values at baseline; thus, this could be an analogic phenomenon. Notably, Jones et al stated that they “recoloured these through a trial and error process of manipulating the color balance and brightness”. We have measured color values from Figure 1 of their manuscript and found significant differences not only in the a* values but also in the L* values, which we know interact with each other. In addition, the redness levels in their visual stimuli were arbitrarily set as pale pink, light pink, dark pink, and red, with no known color distances between them, which may also have affected the attractiveness ratings.

Skin lightness (L* value) had a much lower effect on perceived attractiveness than other color dimensions did, and morphological features were measured. This is in line with the results of studies on facial attractiveness, which indicate that melanin has little effect on perceived attractiveness.^36^ The very small effect of genital skin lightness on attractiveness might indicate that any effect of removing labia pigmentation (genital bleaching) has on genital attractiveness is related only to increased visibility of the skin vasculature. Therefore, methods that increase microcirculation can have similar effects in this context.

Since the absolute length of the labia minora is not easily evaluated under real-life conditions, we used a measure of labia minora visibility (the percentage of the area with visible labia minora vs the whole vulva area) as a more ecologically valid measure of labia size. The results of the current study confirm its major importance for genital aesthetics. Unsurprisingly, labiaplasty is associated with significant patient satisfaction.^17-19^ However, the size of labia minora is normally distributed among young women, and their size has been reported to diminish with age rather than elongate^8^; therefore, in evolutionary terms, one would not expect labia minora length to be a valid cue for age or fertility, which generally translate to attractiveness. Moreover, the chromatic properties of the vulva explained more variance in the attractiveness evaluation than did the morphological features. Therefore, is the loss of labia tissue behind increased aesthetic satisfaction, or other factors may also be involved? A recent study reported a correlation between labia hypertrophy and hyperpigmentation,^10^ which could obscure skin perfusion shown to be a positive predictor of genital attractiveness. Moreover, increased labia redness is common as a result of surgical intervention and scar formation. These color changes may have partially contributed to the increased aesthetic value after labiaplasty. This raises the question of whether labiaplasty should be a first-choice FCGS in patients without functional complaints.

The labia majora apparent volume was significantly correlated with perceived attractiveness; however, the size effect of the labia majora skin texture was twice as significant. The texture of genital skin, also referred to as skin smoothness or turgor, plays an important role in sexual attractiveness in other primate species.^37-39^ Although devising skin texture grading scale we looked for analogies with scales used for sexual swellings in primatological studies,^39^ this scale also clearly reflects the age-related atrophy of labia majora. Our interpretation of these results is that the aesthetic benefit of volumizing FGCSs, such as the lipotransfer of hyaluronic acid filler injection, lies less in creating a certain “attractive” volume than in smoothing the skin surface.

Finally, the presence of a skin crease between the labia majora and the mons pubis was associated with decreased attractiveness. Although the evidence level would be anecdotal, the presence of that crease was notable in our study population, mostly in models that were older and overweight; hence, it could be a cue to either of these factors.

Notably, our final model explained only 58.5% of the variance in genital attractiveness; therefore, in addition to skin color and color contrast, labia majora volume, skin texture, and labia minora visibility are other factors that significantly contribute to genital aesthetics, and further studies in the field of genital aesthetics are needed.

Although majority of the study participants who rated image attractiveness were women, still the study relies on external aesthetic judgments. It remains uncertain how they align with patient satisfaction in FGCS. Additionally, it is unclear if general population and women seeking FGCS share the same aesthetic preferences. Further studies based on patient-reported outcome measures are necessary to validate clinical applicability of our findings. The study design, where both rated models and rating observers were all Caucasian Central Europeans assured that participants rated images they were familiar with, it is unclear, however, if such results are generalizable to individuals of other ethnicities. Previous studies^40^ pertaining the impact of lip vermilion “redness” (considered as a* value of CIELAB color scale) on perceived youthfulness has indicated that faces with increased lip vermilion redness were perceived younger regardless of the cultural origin of the participants and the ethnic origin of the faces (Caucasians, Latin Americans, Chinese, and South African). This analogy indicates that general applicability of our study results is plausible, although further studies including subjects and participants of other ethnicities are necessary.

## CONCLUSIONS

In conclusion, the results of our study confirm, that visibility of labia minora has a major impact on perception of female genital aesthetics in Caucasian women. The apparent volume of labia majora positively predicts perceived genital attractiveness independently of its effect on visual screening of labia minora. The novel finding of this study is that genital skin quality, both in terms of color and texture, significantly contributes to female genital aesthetics. Skin wrinkling and paler skin color, a tell-tale of diminished blood flow, are negative predictors of genital attractiveness. Therefore, we should look beyond just labiaplasty in female genital cosmetic surgery and place more emphasis on the skin color and texture of the labia. The findings of this study provide additional rationale for consideration of non-surgical options, such as laser procedures or peelings as means of improving female genital aesthetics. Additionally, results of this study suggest that cues of obesity may also be discernible in genital area and have a negative effect on genital aesthetics.

## Supplemental Material

This article contains supplemental material located online at https://doi.org/10.1093/asj/sjaf230.

## Supplementary Material

sjaf230_Supplementary_Data
