# Supplementary material for: Think Smooth and Pink: The Role of Skin Color and Texture in Caucasian Female Genital aesthetics
Source: Aesthet Surg J. 2025 Nov 7;46(9):952–8. doi: 10.1093/asj/sjaf230 (PMC13481094; doi:10.1093/asj/sjaf230)
Supplement: Supplementary file 1 [file sjaf230_supplementary_data.zip › 20250829_Supplemental_Table_1.doc]

**Supplemental Table 1**. Pairwise Post Hoc Comparisons for Labia Maiora Skin Texture

|  |  | *t* | *df* | *pHolm* | Cohen’s *d* |
| --- | --- | --- | --- | --- | --- |
| grade 3 (significant atrophy) | grade 1(scrotal) | 5.22 | 896 | < .001 | 1.05 |
|  | grade 2(minimal atrophy) | 2.99 | 896 | .003 | 0.62 |
|  | grade 0 (smooth) | 7.46 | 896 | < .001 | 1.47 |
| grade 1(scrotal) | grade 2(minimal atrophy) | 3.99 | 896 | < .001 | 0.43 |
|  | grade 0 (smooth) | 5.42 | 896 | < .001 | 0.42 |
| grade 2(minimal atrophy) | grade 0 (smooth) | 8.60 | 896 | < .001 | 0.85 |
