# Supplementary material for: Think Smooth and Pink: The Role of Skin Color and Texture in Caucasian Female Genital aesthetics
Source: Aesthet Surg J. 2025 Nov 7;46(9):952–8. doi: 10.1093/asj/sjaf230 (PMC13481094; doi:10.1093/asj/sjaf230)
Supplement: Supplementary file 1 [file sjaf230_supplementary_data.zip › Supplemental_Table_2.doc]

**Supplemental Table** **2.** Pairwise Post Hoc Comparisons for Labia Apparent Volume

|  |  | t | df | pHolm | Cohen’s d |
| --- | --- | --- | --- | --- | --- |
| Grade 3 | Grade 2 | 4.40 | 895 | < .001 | 0.44 |
|  | Grade 1 | 6.28 | 895 | < .001 | 0.50 |
|  | Grade 0 | 6.30 | 895 | < .001 | 0.89 |
| Grade 2 | Grade 1 | 0.60 | 895 | .549 | 0.06 |
|  | Grade 0 | 3.03 | 895 | .007 | 0.45 |
| Grade 1 | Grade 0 | 2.89 | 895 | .008 | 0.40 |

Grade 3 represents highest apparent labia volume
